# Cyanobacterial type I CRISPR-Cas systems: distribution, mechanisms, and genome editing applications

**DOI:** 10.3389/fbioe.2025.1552030

**Published:** 2025-02-27

**Authors:** Yongjiu Zhang, Shuxiao Yang, Xianliang Zheng, Xiaoming Tan

**Affiliations:** ^1^ State Key Laboratory of Biocatalysis and Enzyme Engineering, School of Life Sciences, Hubei University, Wuhan, Hubei, China; ^2^ AngelYeast Co., Ltd., Yichang, Hubei, China; ^3^ National Key Laboratory of Agricultural Microbiology, AngelYeast Co., Ltd., Yichang, Hubei, China

**Keywords:** type I CRISPR-Cas, cyanobacterium, genome editing, synthetic biology, metabolic engineering

## Abstract

Cyanobacteria, renowned for their photosynthetic capabilities, serve as efficient microbial chassis capable of converting carbon dioxide into a spectrum of bio-chemicals. However, conventional genetic manipulation strategies have proven incompatible with the precise and systematic modifications required in the field of cyanobacterial synthetic biology. Here, we present an in-depth analysis of endogenous CRISPR-Cas systems within cyanobacterial genomes, with a particular focus on the Type I systems, which are the most widely distributed. We provide a comprehensive summary of the reported DNA defense mechanisms mediated by cyanobacterial Type I CRISPR-Cas systems and their current applications in genome editing. Furthermore, we offer insights into the future applications of these systems in the context of cyanobacterial genome editing, underscoring their potential to revolutionize synthetic biology approaches.

## 1 Introduction

Cyanobacteria, a phylum of prokaryotic organisms, are capable of fixing carbon dioxide and releasing oxygen into the atmosphere through the process of photosynthesis. As one of the most ancient forms of life on Earth, they have been a dominant force in Earth’s ecosystems for approximately a billion years, markedly enhancing the levels of atmospheric oxygen (Demoulin et al., 2019). In current aquatic ecosystems, cyanobacteria continue to play a pivotal role in the biogeochemical cycling of nutrients across various habitats (Bhardwaj et al., 2024).

Because of their rapid autotrophic growth rate, genetic amenability, and photosynthetic capabilities, cyanobacteria have been considered as a promising photosynthetic chassis for biotechnological applications (Ducat et al., 2011; Angermayr et al., 2015). Metabolic engineering efforts have enabled the biosynthesis of a broad spectrum of products from CO_2_ in cyanobacteria, thereby demonstrating the substantial potential of cyanobacterial photosynthetic biotechnology (Ni et al., 2018). Nonetheless, the low product yields remains a critical barrier to the commercial feasibility of these technologies (Wang et al., 2020).

Systematic metabolic engineering is an effective approach to boost the efficiency of microbial biosynthesis. However, it is challenging to realize genome-scale modifications in cyanobacteria by using conventional genetic manipulation methods which reply on a limited numbers of drug markers. In contrast, CRISPR-Cas dependent genome editing facilitates the targeted modification of genomic loci in a marker-free manner and enables simultaneous modifications across multiple genomic loci, making it an ideal tool for the systematic metabolic engineering of cyanobacteria (Sun et al., 2018). Thus, many efforts have been invested in the introduction of CRISPR-Cas9 or CRISPR-Cas12a genome editing systems for cyanobacteria, as summarized in some recent reviews (Carroll et al., 2018; Vijay et al., 2019; Pattharaprachayakul et al., 2020; Dong et al., 2024).

In recent years, the utilization of native type I CRISPR-Cas systems for genome editing has emerged as a promising strategy in prokaryotes (Zheng et al., 2020; Xu et al., 2021). Unlike the widely employed exogenous Cas9 and Cas12a effectors, endogenously encoded Cas effectors in type I systems do not require additional over-expression and exhibit better compatibility with the host (Zheng et al., 2019; Du et al., 2022). Notably, the majority of cyanobacterial genomes encode endogenous CRISPR-Cas systems, which serve as immune systems against foreign DNA invasions (Doron et al., 2018; Strecker et al., 2019; Ziemann et al., 2023). The widespread distribution of type I CRISPR-Cas systems in cyanobacteria (Makarova et al., 2019; Pattharaprachayakul et al., 2020) holds significant potential for endogenous CRISPR-based genome engineering.

Recently, some studies have been performed to elucidate the adaptation and interference mechanisms of these native CRISPR-Cas systems in cyanobacteria (Jesser et al., 2019; McBride et al., 2020; Schwartz et al., 2022). Furthermore, several cyanobacterial CRISPR-Cas systems have been harnessed for genetic manipulations in mammalian cells or higher plants (Keishi Osakabe, 2020; Osakabe et al., 2021). We have successfully reprogrammed the native Type I CRISPR-Cas system of *Synechococcus* sp. PCC 7002 to target its own genome, thereby achieving marker-less gene deletions (Yang et al., 2024).

In this review, we present a comprehensive analysis of the native CRISPR-Cas systems within cyanobacteria. Our examination encompasses the prevalence of these systems across cyanobacterial genomes. Additionally, we summarize the underlying immune mechanisms of type I CRISPR-Cas systems, their current application in genome editing, and offer insights into the potential future use of type I CRISPR-Cas systems in cyanobacterial biotechnology.

## 2 Native CRISPR-Cas systems of cyanobacteria

### 2.1 Distribution of CRISPR-Cas systems in cyanobacterial genomes

In an analysis of 126 cyanobacterial genomes, it was predicted that the majority of these genomes harbored the CRISPR-Cas system (Cai et al., 2013). Additionally, a prevalence of CRISPR-Cas systems was identified in 171 genomes of multicellular cyanobacteria (Hou et al., 2018). To elucidate the distribution of CRISPR-Cas systems within cyanobacteria, we conducted a re-analysis of endogenous CRISPR-Cas systems in publicly available complete cyanobacterial genomes using the CRISPRCasTyper pipeline (Russel et al., 2020).

**FIGURE 1:**
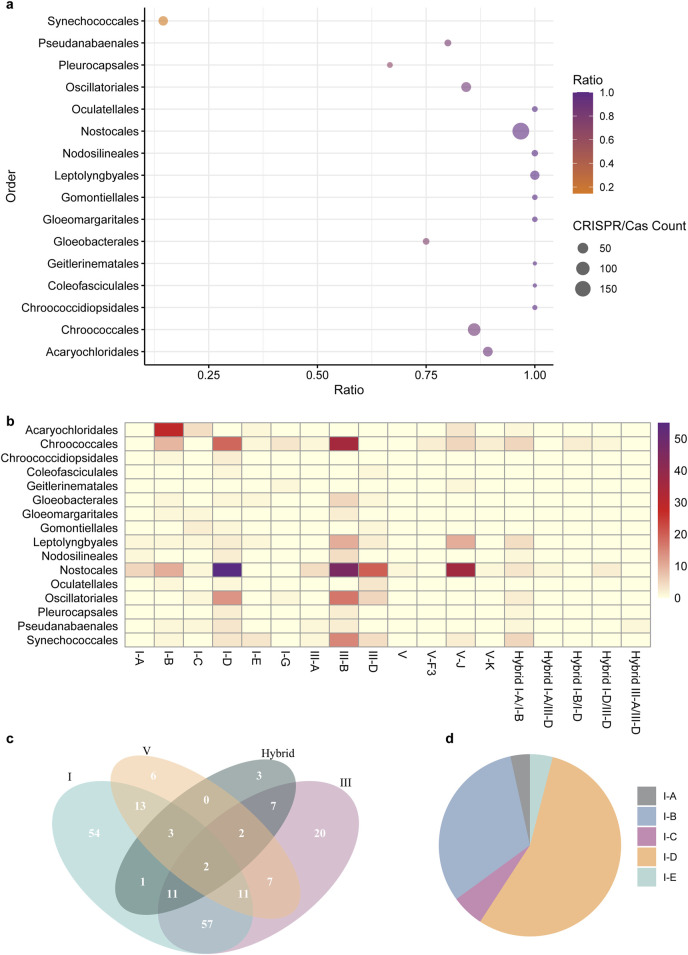
Distribution and prevalence of CRISPR-Cas Systems across cyanobacterial Orders. **(A)** The scatter plot presents the numbers of CRISPR-Cas systems across different cyanobacterial orders and the ratio of genomes containing the system to the total number of genomes in the specific orders. The x-axis represents the ratio, while the y-axis lists the cyanobacterial orders. The size of each point corresponds to the count of CRISPR-Cas systems within that order, with larger points indicating a higher count, as detailed in the legend. The color gradient from orange to purple corresponds the ratio of genomes with CRISPR-Cas systems. **(B)** The heatmap visualizes the abundance of different types of CRISPR-Cas systems across various cyanobacterial orders. The rows represent distinct CRISPR-Cas types (I-A, I-B, I-C, etc.), while the columns list the cyanobacterial orders. The color intensity corresponds to the number of CRISPR-Cas systems identified within each order, as indicated by the color legend. **(C)** The Venn diagram illustrates the co-existence of different CRISPR-Cas system types: I, III, and V, along with hybrid systems, in cyanobacterial genomes. Each circle represents a unique system type, with the numbers inside the overlapping areas indicating the count of genomes containing both of the systems. **(D)** The pie chart illustrates the proportion of five distinct subtypes of cyanobacterial Type I CRIPSR-Cas system. Each color corresponds to a specific subtype.

We retrieved 335 complete cyanobacterial genomes from the NCBI Genome database (29 October 2024). Among the 315 unique genomes, at least one CRISPR-Cas system was identified in 197 genomes, representing 62.5% of the cyanobacterial genomes examined (Supplementary Table S1). The distribution of CRISPR-Cas systems was found to be significantly variable across different orders of cyanobacteria. A substantial majority (84.7%) of cyanobacteria lacking CRISPR-Cas systems were classified under the order Synechococcales. Upon excluding the Synechococcales order, 180 (90.9%) of the remaining 198 cyanobacterial genomes were predicted to contain the CRISPR-Cas system. Notably, nearly all genomes from the Nostocales (96.7%) and Leptolyngbyales (100%) orders, as well as over 80% of genomes from the Acaryochloridales, Chroococcales, and Oscillatoriales, were found to contain CRISPR-Cas systems (Figure 1A).

All cyanobacterial CRISPR-Cas candidates can be categorized into two major classes with three types, including types I and III in the Class 1, and types V in the Class 2 (Figure 1B). Type I, III, V and certain hybrid CRISPR-Cas systems were found in 152, 117, 44 and 29 cyanobacterial genomes, respectively, accounting for 48.3%, 37.1%, 14.0% and 9.2% of the surveyed genomes. Notably, type I systems were found in all 16 orders of cyanobacteria (Figure 1B), indicating that type I CRIPSR-Cas systems are most prevalent in cyanobacteria.

Additionally, some cyanobacterial genomes harbor more than one type of CRISPR-Cas. Eighty-one cyanobacterial genomes (25.7%) were found to contain both type 1 and III systems, while thirteen genomes contained all three types (type I, III and V) of CRISPR-Cas systems (Figure 1C). Among the identified type I systems, the type I-D system is the most prevalent, accounting for 63.2% of the surveyed genomes, followed by I-B, I-C, I-A, and I-E (Figure 1D).

### 2.2 DNA defense mechanisms mediated by cyanobacterial type I CRISPR-Cas systems

Normally, the natural type I CRISPR-Cas system’s response to invading DNA can be categorized into three distinct stages: adaptation, processing of CRISPR RNA (crRNA), and interference (Zheng et al., 2020). *Synechocystis* sp. PCC 6803 (hereafter Syn6803) was chosen as a model for most fundamental researches on cyanobacterial type I system.

**FIGURE 2:**
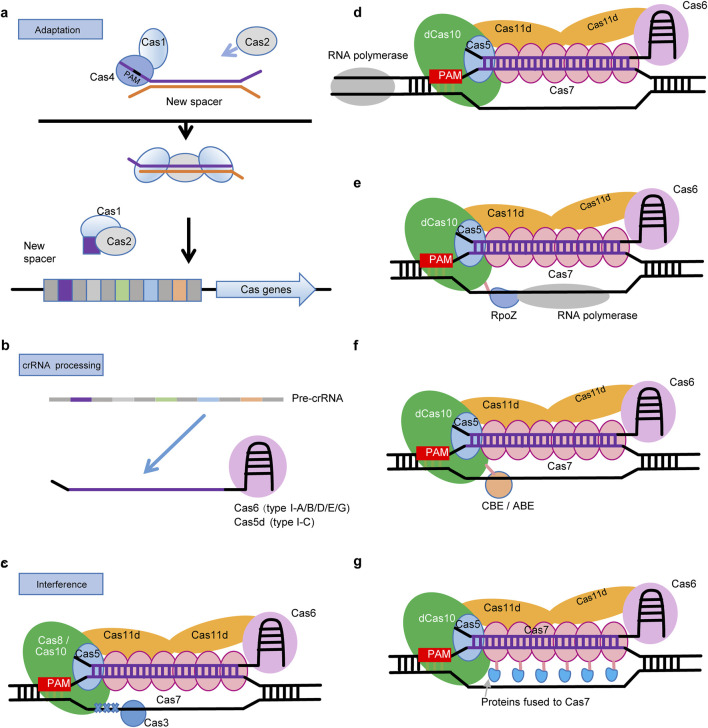
Mechanisms and applications of cyanobacterial type I CRISPR-Cas in genome editing. **(A)** Adaptation: Cas1 and Cas2 proteins capture a short sequence from an exogenous DNA and integrate it into the CRISPR array as a new spacer. In some type I systems, Cas4 is also required for the selection of spacers. **(B)** crRNA Processing: Transcription of the CRISPR array generates pre-crRNA with multiple hairpin structures. Cas5d (type I-C) or Cas6 (other type I systems) process the pre-crRNA by binding to its 3′end. **(C)** Interference: Subsequently, the Cas5 binds to the 5′-handle, and the Cas7 subunit associates with the crRNA to form the structural backbone of the crRNA. In most type I systems, Cas8 binds to Cas5 and recognizes the PAM, Cas3 then initiates cleavage through its nuclease activity. In contrast, the type I-D system lacks Cas8, with Cas10 responsible for both PAM recognition and DNA cleavage. Here, Cas3 exhibits only helicase activity. **(D)** CRISPRi: In cyanobacterial type I systems, when Cas3 or Cas10 is inactivated, it can bind but not cleave DNA, preventing RNA polymerase progression and resulting in gene silencing. **(E)** CRISPRa: Inactivated Cas10 (type I-D) or Cas3 (other type I systems) is fused with the ω-subunit (RpoZ) of RNA polymerase. The latter recruits the core complex of RNA polymerase, thus activating the expression of targeted gene. **(F)** Base Editing: Inactivated Cas10 (type I-D) or Cas3 (other type I systems), without cleavage activity, fused with a cytosine deaminase (CBE) or an adenosine (ABE), targets specific sites for base editing. **(G)** Besides deficient nucleases, Cas7 should be another choice for fusion expression of RpoZ or deaminases.

During the adaptation stage, a short DNA fragment is captured, processed and integrated into the host’s CRISPR arrays (Figure 2A). The Cas1-Cas2 complex is universally implicated in this process. Besides, Cas4, encoded by a gene typically located adjacent to *cas1* and *cas2*, is also required for the selection of spacers with a specific protospacer adjacent motif (PAM) in certain type I systems (Kieper et al., 2018; Lee et al., 2018; Zhang et al., 2019). A recent structural analysis of the type I-D CRISPR-Cas in Syn6803 showed a two-step assembly mechanism involving these three Cas proteins during the adaptation stage (Wu et al., 2021). Initially, Cas4 forms a stable complex with Cas1, which processed prespacer precursors into their mature form in a PAM-dependent manner. This maturation of prespacers leads to the disassembly of the Cas1-Cas4-prepacer complex and facilitates the assembly of Cas1–Cas2–prespacer complex. Finally, the prespacer, now associated with the Cas1-Cas2 complex, is integrated into the host’s CRISPR array as a new spacer (Wu et al., 2021).

In the crRNA processing stage, mature crRNAs are cleaved from pre-crRNA which results from the transcription of CRISPR array (Figure 2B). CRISPR repeats in the pre-cRNA form stable stem-loop structures that are recognized and processed by a RNA endonuclease. Type I-C systems use a unique Cas5 variant, Cas5d (Punetha et al., 2014), while the rest of type I systems use Cas6 for crRNA processing. Each subtype of type I CRISPR-Cas has its own specific stem-loop structures, with variations in stem and loop sizes and sequences. After crRNA processing, Cas6 proteins remain bound to the 3′hairpin structure of the mature crRNA (Zheng et al., 2020). Base on both *in vivo* and *in vitro* analyses, Cas6-1 is found to be a specialized RNA endonuclease critical for the maturation of crRNA in the type I-D CRISPR-Cas of Syn6803, exhibiting a single turnover mechanism and requiring a conserved histidine residue for enzymatic activity and positive residues for RNA binding (Scholz et al., 2013; Reimann et al., 2016; Jesser et al., 2019).

In the interference phase, Cas6-crRNA assembles with Cas7, Cas5, and Cas8 to from a CRISPR associated complex for antiviral defense (Cascade) (Figure 2C). In a typical type I Cascade complex (Lu et al., 2024), multiple Cas7 proteins binds around the central part of crRNA, while Cas5 and Cas6 bind to 5′ and 3′-end of crRNA respectively. In most type I systems, a large subunit Cas8 which is normally located adjacent to Cas5 is responsible for recognizing PAM sequences. Besides, the C-terminus of Cas8 and several small subunits (SSU) constitute the inner “belly” of Cascade.

In response to invading DNA, the Cascade complex, guided by base pairing between the embedded crRNA and protospacer, selectively targets invading DNA molecules, thereby forming an R-loop structure. The binding of dsDNA to the Cascade complex induces a conformational change, facilitating the recruitment of Cas3 which is usually composed of an N-terminal HD phosphohydrolase domain and a C-terminal helicase domain (Sinkunas et al., 2011). Finally, Cas3 nicks and then degrades the invading DNA in the presence of ATP.

However, in the cyanobacterial type I-D system, Cas3d lacks the HD nuclease domain and Cas8 is absent. Instead, Cas10d, which harbors a domain of the nuclease, was proved to be responsible for DNA cleavage (Osakabe et al., 2021) and also compensates for the roles of Cas8, mediating PAM recognition instead (Schwartz et al., 2022). Furthermore, the type I-D Cascade incorporates Cas11d small subunits that are derived from an alternative translation initiation site within Cas10d. Cas11d is essential for the specific binding of the I-D Cascade to target double-stranded DNA (dsDNA), as demonstrated by the significantly reduced DNA-binding capacity of the complex lacking Cas11d. And the structure of the I-D Cascade, featuring a helical backbone of Cas7d subunits and a boot-shaped Cas10d large subunit, aligns more closely with the architecture of Type III systems rather than the more curved Cas7 backbone and smaller Cas8 large subunits typical of other Type I systems (McBride et al., 2020).

### 2.3 Applications of cyanobacterial type I systems in genome editing

Cas9 and Cas12a, derived from type II and V CRISPR-Cas systems, are the most widely utilized CRISPR-associated (Cas) effectors for genome editing. Despite their popularity, type I CRISPR-Cas systems are more prevalent in bacteria and archaea (Makarova et al., 2019) and exhibit a more aggressive interaction with DNA targets, which enables them with a distinct advantage in facilitating large-fragment deletions within host genomes (Cameron et al., 2019; Morisaka et al., 2019; Li J. et al., 2024). Up to now, only a limited number of cyanobacterial type I systems have been employed for genome editing in heterologous hosts. Notably, the type I-B system from *Synechocystis* sp. PCC 6714 has been successfully utilized for genome editing in human T cells, underscoring its potential in human genome editing. This system is characterized by its ability to induce long-spectrum, unidirectional deletions within the human T cells (Lu et al., 2024). Furthermore, the Cascasde of type I-D CRISPR-Cas system, comprising five Cas proteins (Cas3, 5, 6, 7, and 10) from *Microcystis aeruginosa*, has been heterologously expressed in mammalian cells for targeted genome editing, enabling the introduction of both small deletions and bidirectional large-fragment deletions ranging from 2.5 to 18.5 kb (Osakabe et al., 2021). This system has also demonstrated the capacity to induce short indels and bi-directional long-range deletions in tomato cells (Keishi Osakabe, 2020). Recently, we have successfully developed a genome editing tool based on the endogenous type I-D CRISPR-Cas system of *Synechococcus* sp. PCC 7002, enabling precise genetic modifications in this organism. Utilizing this tool, we deleted the *glgA1* gene and created a double mutant of the *glgA1* and *glgA2* genes, thereby demonstrating the system’s capability for targeted genome editing. Additionally, we exhibited the tool’s ability to knock out large genomic fragments and to cure the endogenous pAQ5 plasmid, highlighting its potential for genome streamlining (Yang et al., 2024).

### 2.4 Future perspectives

These reports prove the potential of endogenous type I CRISPR-Cas systems for precise genome editing. Notably, these native systems alleviate concerns regarding cell toxicity associated with Cas effectors, which often limit the application of the widely used exogenous CRISPR-Cas9 systems (Wendt et al., 2016). Given the prevalence of type I systems in cyanobacterial genomes (Supplementary Table S1), these native systems hold promise for repurposing as efficient genome editing tools for their respective cyanobacterial hosts.

In contrast to genome editing via DNA double-strand breaks (DSB) induced by Cas nucleases, several CRISPR-derived technologies, including CRISPR interference (CRISPRi) (Qi et al., 2013), CRISPR activation (CRISPRa) (Maeder et al., 2013), and base editing (BE) (Komor et al., 2016), can modulate transcriptional levels of target genes or introduce point mutations without DSB. These technologies typically rely on nuclease-deficient Cas9 (dCas9 or nCas9), which can bind but not cleave the double-strand of target DNA. To date, all reports on the application of these strategies in cyanobacteria (Gordon et al., 2016; Huang et al., 2016; Yao et al., 2016; Bourgade et al., 2024; Li X.-D. et al., 2024; Li X. et al., 2024) are based on heterologous type II or V Cas effectors.

To implement these strategies in cyanobacteria using endogenous type I CRISPR-Cas systems, it is necessary to modify cyanobacterial genomes to inactivate the key nuclease subunit of Cascade, specifically Cas10 in type I-D (Osakabe et al., 2021) and Cas3 in other type I systems (Zheng et al., 2019). The binding of nuclease-deficient Cascade impedes the passage of RNA polymerase, thereby achieving highly specific inhibition of gene transcription (CRISPRi) in cyanobacteria (Figure 2D). To enhance the expression levels of specific genes in cyanobacteria, the inactivated nucleases can be further modified to fuse with the omega subunit of RNA polymerase (RpoZ), which recruits the host’s RNA polymerase core enzymes. Thus, RNA polymerase will be guided to the specific gene region, enhancing the transcription of target genes (Figure 2E). For precise nucleotide substitution in specific genomic regions in cyanobacteria, the inactivated nucleases can be fused with a deaminase that mediates C-to-T (CBE) or A-to-G (ABE) substitutions (Figure 2F). It is worthy to note that, Cas7 can also serve as an alternative for fusing RpoZ or deaminases (Figure 2G), in addition to inactivated nucleases, considering the multiple copies of Cas7 within the Cascade complex of type I systems (Guo et al., 2024).

## 3 Discussion

Cyanobacteria have been recognized as a promising class of photosynthetic chassis microorganisms that are able to convert CO_2_ to bio-chemicals. To improve CO_2_ conversion efficiency by recombinant cyanobacteria, efficient genetic tools compatible with systematic genetic modifications are essential. In this review, we have analyzed the distribution of CRISPR-Cas systems in completed cyanobacterial genomes to date, summarized the current understanding of the adapted immune mechanisms of native type I systems, and provided future perspectives on the application of these native systems for cyanobacterial genome editing.

The cyanobacteria within the order Synechococcales that lack CRISPR-Cas systems are primarily from the marine families Prochlorococcaceae and Synechococcaceae, which is consistent with the previous survey (Cai et al., 2013). In contrast, seven cyanobacteria from the Merismopediaceae family, which also belong to the Synechococcales order, such as the well-known model *Synechocystis* sp. PCC 6803 and PCC 6714, do possess CRISPR-Cas systems (Supplementary Table S1), suggesting a divergence within the Synechococcales order regarding the presence of CRISPR-Cas system. Furthermore, a recent survey highlighted a strong association between CRISPR-Cas systems and cyanobacteria thriving in eutrophic and hyper-eutrophic conditions (Park et al., 2024), indicating that the presence or absence of these systems may depend on the ecological niche and evolutionary pressures faced by the cyanobacteria.

Compared to the foreign CRISPR-Cas9 system, endogenous type I CRISPR-Cas systems offer advantages in terms of cell compatibility, long-range editing capabilities, and target specificity (Yoshimi and Mashimo, 2022), making them suitable as species-specific genetic tools for cyanobacterial species harboring these endogenous systems. However, several challenges remain to be addressed to fully realize their potential. First, for some endogenous type I CRISPR-Cas systems with PAM sequences yet to be identified, it is essential to characterize their PAM sequence requirements first. Second, successful gene editing depends critically on efficient gRNA delivery and expression. Both transcriptional levels of gRNA and its structural parameters (sequence design and length optimization) need to be systematically evaluated. Third, due to the polyploid nature of cyanobacterial genomes, careful screening for homozygous mutant strains is essential to ensure both genotypic and phenotypic stability in the edited strains. In addition, further fundamental research on native cyanobacterial CRISPR-Cas systems is necessary, including their off-target effects and responses to foreign DNA, to optimize these endogenous tools for future applications.
